# Regime shift in fish assemblage structure in the Yangtze River following construction of the Three Gorges Dam

**DOI:** 10.1038/s41598-019-38993-x

**Published:** 2019-03-12

**Authors:** Xin Gao, Masami Fujiwara, Kirk O. Winemiller, Pengcheng Lin, Mingzheng Li, Huanzhang Liu

**Affiliations:** 1 Key Laboratory of Aquatic Biodiversity and Conservation of Chinese Academy of Sciences, Institute of Hydrobiology, Chinese Academy of Sciences, Wuhan, Hubei China; 2 Department of Wildlife and Fisheries Sciences, Texas A&M University, College Station, TX 77843-2258 USA

## Abstract

Dams have well-documented ecological impacts on downstream river segments; however, long-term impacts of river impoundment have rarely been investigated in upstream reaches. Using data from long-term standardized surveys, we analyzed temporal changes in fish assemblages in the Yangtze River upstream of the Three Gorges Dam (TGD) before, during and after its construction. Our analysis indicated fish assemblage regime shifts in the two closer reaches in 2008, in accordance with the filling to 172.5 m in 2008; and in the other reach, farthest from the TGD, in 2011, indicating timing of the effects being related to distance. These shifts were evident in relative abundance of native fish species rather than non-native species and have altered community structures and functional groups. Relative abundance of the lotic guilds declined in the two closer reaches, but increased in the farthest. Invertivores declined, but piscivores and opportunistic life-history strategists increased in all reaches. We conclude that construction of TGD had led to significant changes in species distributions influenced by species functional traits. Our findings emphasize the need for long-term monitoring of fish assemblages before and after dam construction in order to understand ecological responses to hydrological changes for effective resource management in regulated rivers.

## Introduction

Dams and reservoirs create economic and social benefits, but also impact riverine ecosystems in multiple ways^1–7^. These impacts include drowning of channel and riparian habitats, reduction in dissolved oxygen concentrations within the impounded zone, and alteration of hydrology, thermal regime, and sediment and nutrient dynamics in downstream regions^8–11^. Dams and reservoirs also block migration routes for native fishes^12–15^. Consequently, fish abundance and diversity are often severely affected by construction of dams and reservoirs^16^. Impoundments alter instream and riparian habitat^12–14^, which, in turn, affect aquatic organisms both upstream and downstream from reservoirs^17^. Although the effects of impoundments on downstream fish assemblages have been well documented^18–24^, their effects on upstream fish assemblages remain poorly understood^25,26^ and are sometimes assumed to be limited^27^.

Three Gorges Dam (TGD), which is located near the city of Yichang in the middle reaches of the Yangtze River in central China, is one of the largest dams in the world. The Three Gorges Reservoir (TGR) is 1,080 km^2^ in surface area and can store up to 3.93 × 10^10^ m^3^ of water. The TGR was filled in three stages. The first stage raised the water level to 135 m ASL (above sea level) in 2003, and the second stage raised the level to 156 m ASL in 2006. The reservoir was filled to 172.5 m ASL in 2008 and then 175 m ASL in 2010. The water level is currently regulated. It is reduced to 145 m in a wet season from May to September for flood control and is raised to 175 m in the other seasons for power generation and shipping. Environmental impacts of the TGR on downstream reaches of the Yangtze River have been documented^28,29^. These effects include eutrophication, phytoplankton blooms, changes in the structure of macroinvertebrate community, and reduction in the natural reproduction of endangered as well as commercially important fishes, such as Chinese sturgeon (*Acipenser sinensis*) and major Chinese carps^30–35^.

At the same time, the construction of the TGD/TGR can be exploited as a massive manipulative field experiment for testing ideas about aquatic community responses to a major disturbance^36^. However, there have been few studies of the TGD/TGR on fish assemblages in upper reaches of the Yangtze River to date^37–39^. Here, we examine variation in fish assemblage structure in reaches above the TGR by analyzing standardized survey data for an 18-year time series spanning periods before and after completion of the TGD.

Kirkman *et al*.^40^ categorized temporal ecological changes into three types: inter-annual variation in structure, gradual temporal change, and sudden change representing a regime shift. A regime shift is defined as an evident, sudden, and temporally persistent alteration in the state, structure, or function of an ecological system^41–43^. The number of studies investigating regime shifts in aquatic systems has increased in recent years. Most studies have focused on the dynamics of plankton, fish and food webs^44–48^ and sought to identify key drivers of regime shifts, such as climate change, overexploitation, exotic species introduction, and alteration of hydrology^45,49–52^. We hypothesized that the impoundment of the Yangtze River could have caused a regime shift in the structure of fish assemblages in reaches upstream from the TGR. To test this hypothesis, we analyzed a time-series of species-specific data for fish abundance from standardized surveys conducted at multiple sites between 1997 and 2015. To gain insights about potential mechanisms driving temporal patterns, we analyzed fish assemblages based on both taxonomic and functional structures.

## Results

**Figure 1:**
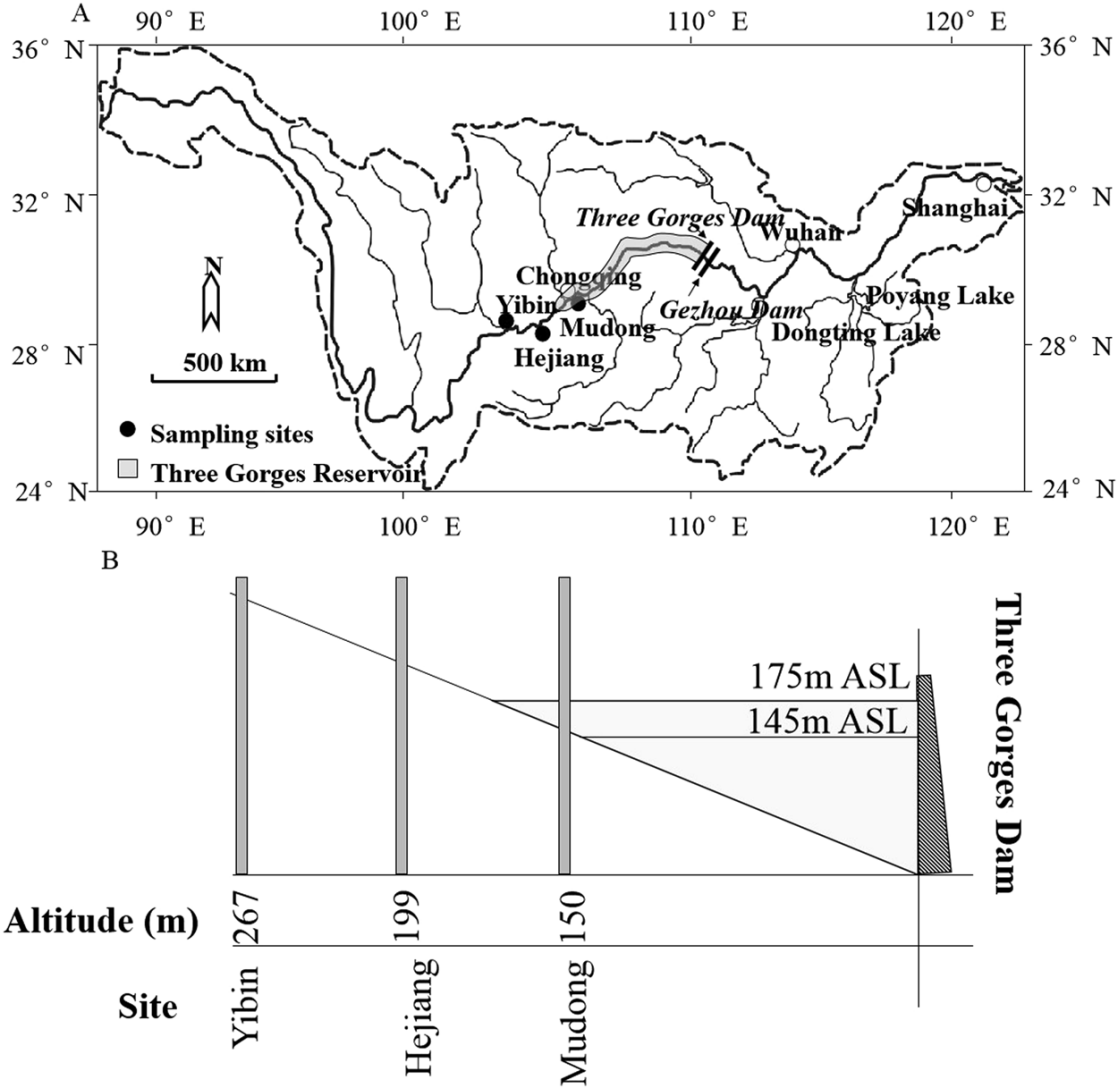
(**A**) Map of Yangtze River basin and fish survey sites; (**B**) Operational water levels of the Three Gorges Dam and elevations at the three survey sites.

Surveys conducted in the three reaches (Yibin, Heijiang, and Mudong; Fig. 1) over the entire study period yielded 498,023 specimens representing 150 fish species in 24 families and 10 orders. The most abundant species belonged to the order Cypriniformes (70.7% of total abundance). The two most abundant species were *Coreius guichenoti* (Cyprinidae, Cypriniformes, 27.6% of total abundance) and *Pelteobagrus vachelli* (Bagridae, Siluriformes, 15.6% of total abundance). Twelve non-native species, namely *Ictalurus punctatus*, *Tinca tinca*, *Piaractus brachypomus*, *Clarias gariepinus*, *Micropterus salmoides*, *Oreochromis sp*., *Lucioperca lucioperca*, *Protosalanx hyalocranius*, *Megalobrama skolkovii*, *Megalobrama amblycephala* and *Acipenser schrenckii*, and hybrid sturgeon were collected and accounted for 0.03% of the total abundance. Fish abundance in samples was highest in Mudong reach and lowest in Yibin reach. The most abundant species within each reach were as follows: Yibin -*Pelteobagrus vachelli* and *Coreius guichenoti*; Hejiang - *Coreius guichenoti* and *Pelteobagrus vachelli*; and Mudong - *Coreius guichenoti* and *Coreius heterodon*.

**Figure 2:**
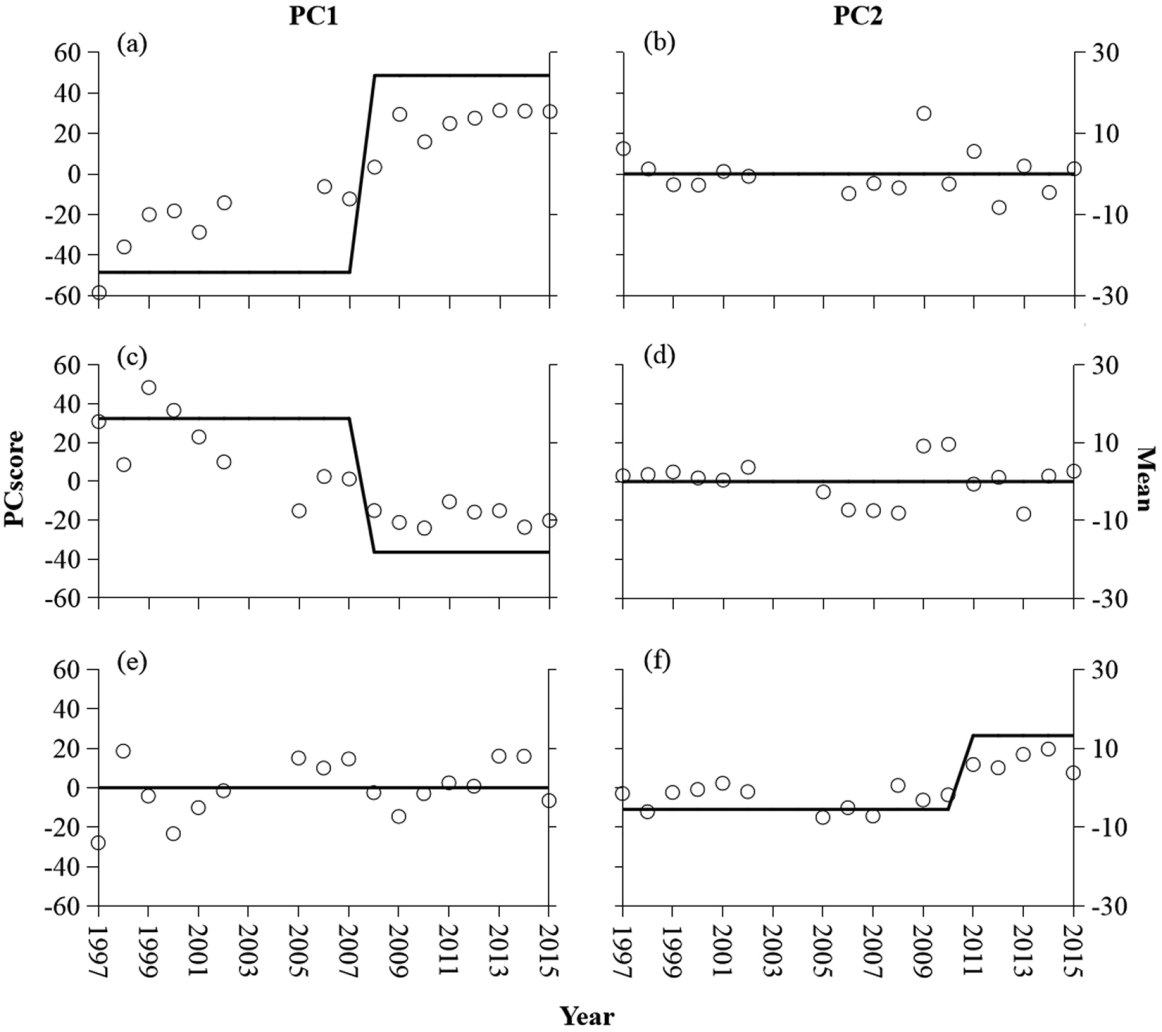
Regime shifts derived from STARS analysis of the time series for the first and second axis from principal components analysis of fish assemblage data: Mudong (**a,b**), Hejiang (**c,d**), and Yibin (**e,f**).

**Figure 3:**
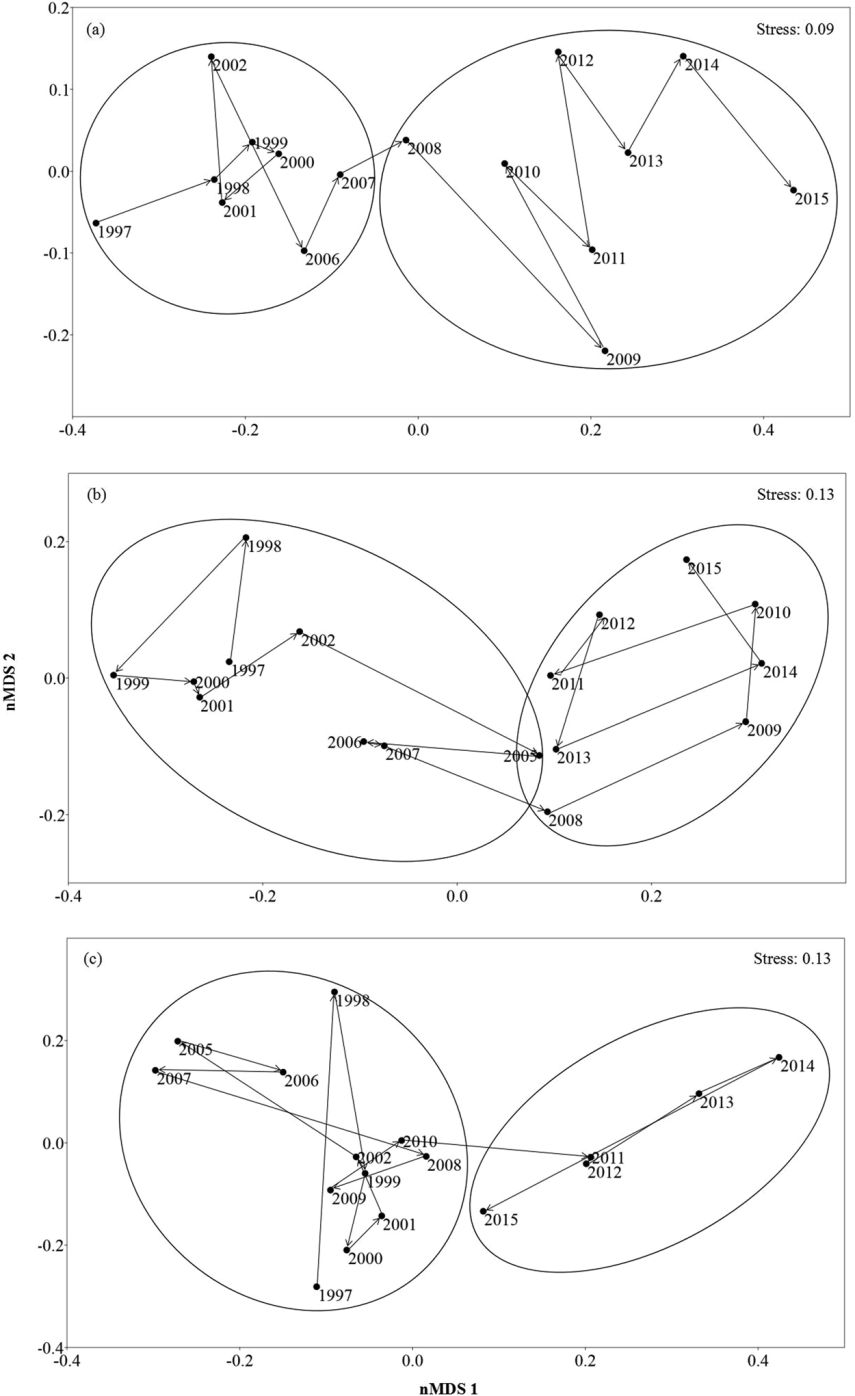
Non-metric multidimensional scaling (nMDS) ordination plot depicting variation in fish assemblage structure at Mudong (**a**), Hejiang (**b**), and Yibin (**c**) reaches located upstream from the Three Gorges Reservoir.

The regime-shift detection analysis revealed that fish assemblages underwent abrupt shifts in the three reaches during the period 1997–2015 (Fig. 2). Based on the first principal component (PC1, representing the dominant gradient of assemblage variation based on species abundance), the significant change in fish assemblage structure occurred in 2008 for Mudong and Hejiang (*P* < 0.001; Fig. 2a,c). The second principal component (PC2, representing a secondary gradient of assemblage variation) revealed significant change in the Yibin fish assemblage in 2011 (*P* < 0.001) (Fig. 2f). Ordination by non-metric multidimensional scaling (NMDS) resulted in six assemblage clusters associated with river reaches and pre- and post-shift periods (stress value: Mudong = 0.09, Hejiang = 0.13, Yibin = 0.13, Fig. 3). Permutational multivariate analysis of variance (PERMANOVA) demonstrated that fish assemblages of all three reaches differed significantly between pre- and post-regime periods (*P* < 0.001). The temporal stability of fish assemblages was greater after the regime shift than before the shift in the Mudong and Yibin reaches, but stability was lower in the Hejiang reach after the regime shift (Table 1).

**Table 1:** Community stability index in Mudong, Hejiang, Yibin reaches before and after regime shifts.

Reaches	Year	Stability index
Mudong	1997–2007	0.75
	2008–2015	2.82
Hejiang	1997–2007	2.56
	2008–2015	2.19
Yibing	1997–2010	2.1
	2011–2015	2.87

Three species diversity indices, Simpson’s, Shannon-Wiener, and Buzas & Gibson’s evenness, increased after the regime shift in the three reaches (Table 2). Changes in the diversity indices between pre- and post-shift were significant in Mudong reach (*P* < 0.05) (Table 2). The number of species increased from 91 to 113 in Mudong reach and from 97 to 124 in Hejiang reach, and the number of species decreased from 82 to 72 in Yibin reach after the regime shift (Table S1). The number of non-native fish species increased after the shift in all three reaches (Table S1), but native fishes dominated fish assemblages in each reaches both before and after the regime shift. These dominant native species included *Coreius guichenoti*, *Pelteobagrus vachelli*, *Coreius heterokon*, and *Rhinogobio cylindricus* (Table 3). Results from the similarity percentage (SIMPER) analysis showed 68.4% (Mudong), 63.8% (Hejiang), and 44.3% (Yibin) dissimilarity in overall assemblage composition between pre- and post-regime shift periods. In Mudong and Hejiang reaches, differences were strongly influenced by reduced relative abundance of *Coreius guichenoti* during the post-shift period (Table 3). In Yibin reach, *Pelteobagrus vachelli* and *Coreius guichenoti* were less abundant during the post-shift period (Table 3). The relative abundance of several small cyprinids and cobitids, such as *Squalidus argentatus*, *Botia superciliaris*, and *Xenophysogobio boulengeri*, increased in the reaches after the regime shift.

**Table 2:** Comparison of diversity indices for fish assemblages in Mudong, Hejiang, Yibin reaches of the Yangtze River before and after regime shifts.

Diversity index		Simpson’s index	Shannon-Wiener index	Buzas & Gibson’s evenness index
Mudong	pre-shift (1997–2007)	0.65 ± 0.07*	1.57 ± 0.16*	0.12 ± 0.01*
	post-shift (2008–2015)	0.875 ± 0.01*	2.62 ± 0.09*	0.20 ± 0.01*
Hejiang	pre-shift (1997–2007)	0.79 ± 0.04	2.25 ± 0.15*	0.20 ± 0.03
	post-shift (2008–2015)	0.89 ± 0.01	2.87 ± 0.06*	0.22 ± 0.01
Yibin	pre-shift (1997–2011)	0.78 ± 0.03	2.20 ± 0.09	0.22 ± 0.015
	post-shift (2012–2015)	0.855 ± 0.01	2.48 ± 0.03	0.26 ± 0.02

^*^P < 0.05 based on the Student t-test.

**Table 3:** SIMPER results identifying species contributing the most to differences in assemblage structures before and after regime shifts in three reaches of the Yangtze River upstream from the TGR.

Mudong				Hejiang				Yibin			
Species	Mean abundance percentage (%)		Dissimilarity (%)	Species	Mean abundance percentage (%)		Dissimilarity (%)	Species	Mean abundance percentage (%)		Dissimilarity (%)
	Pre-shift	Post-shift			Pre-shift	Post-shift			Pre-shift	Post-shift	
*Coreius guichenoti*	51.2	7.6	31.9	*Coreius guichenoti*	34.4	5.1	23.2	*Saurogobio dabryi*	39.2	28.6	16.7
*Saurogobio dabryi*	0.2	13.6	9.8	*Squalidus argentatus*	2.5	15.7	10.6	*Coreius guichenoti*	14.8	4.5	12.3
*Rhinogobio cylindricus*	4.6	15.2	8.2	*Botia superciliaris*	5.3	10.5	8.1	*Xenophysogobio boulengeri*	5.4	13.6	9.9
*Coreius heterokon*	12.7	9.9	5.9	*Pelteobagrus vachelli*	13.8	12.8	6.5	*Botia superciliaris*	2.2	8.6	8.2
*Pelteobagrus vachelli*	12.2	8.8	5.3	*Saurogobio dabryi*	4.8	10.1	6.2	*Jinshaia sinensis*	1.9	7.1	5.9

**Figure 4:**
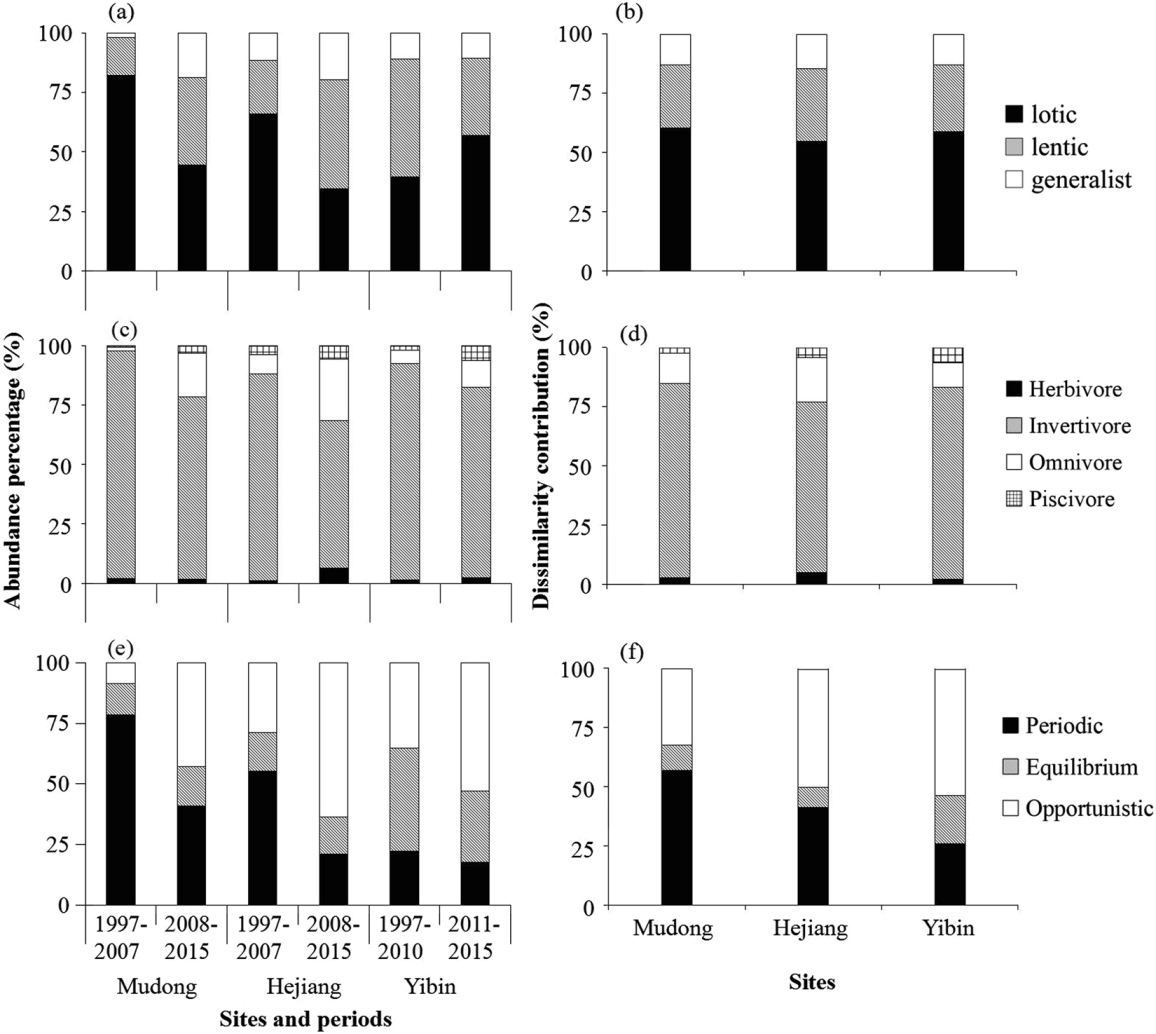
Variation in the relative abundance of functional groups (**a**: habitat, **c**: trophic, **e**: life history), and results of SIMPER analysis (% dissimilarity contribution) for functional groups (**b**: habitat, **d**: trophic, **f**: life history) in Mudong, Hejiang, Yibin reaches before and after regime shifts.

Noticeable changes in functional composition accompanied the regime shift in fish assemblage structure in each reach (Fig. 4). In all three reaches, lotic-adapted invertivores with opportunistic life history strategies contributed most to overall dissimilarity before and after the shift. In the Mudong reach, reduced abundance of fishes with periodic life history strategies also contributed to temporal differences in functional assemblage structure (Fig. 4b,d,f). After the regime shift, the relative abundance of lotic-adapted fishes declined by 45.7% and 47.5% in Mudong and Hejiang reaches, respectively, but increased by 44.6% in the Yibin reach (Fig. 4a). The relative abundance of invertivores declined by 19.6%, 28.4%, and 12.1% whereas piscivores increased by 357.5%, 50.6%, 214.9% in Mudong, Hejiang, and Yibin, respectively (Fig. 4c). Fishes with periodic life history strategies declined by 47.9%, 61.8%, 20.7%, and those with opportunistic strategies increased by 395.1%, 120.8%, 50.7% in Mudong, Hejiang, and Yibin, respectively (Fig. 4e).

## Discussion

Fish assemblages in three reaches of the upper Yangtze River underwent significant regime shifts during or soon after the completion of the TGD and filling of its reservoir. This regime shift occurred in 2008 in Mudong and Hejiang reaches (the locations closer to the reservoir) during initial filling of the TGR to a water level of 172.5 m, and occurred in 2011 in the Yibin reach after the TGR water level was raised to 175 m. Our results suggest that this impoundment affected fish ecology not only within the reservoir, but also in upstream reaches that were not impounded. The delay in the response in the Yibin reach was likely due to its more distant location upstream from the reservoir. Franssen and Tobler^25^ similarly found that there were significant shifts in fish assemblages above a reservoir in Oklahoma, USA, and these shifts were mainly caused by reduced abundance of fluvial specialists and greater abundance of habitat generalists such as mosquitofish (*Gambusia affinis*) and largemouth bass (*Micropterus salmoides*). Falke and Gido^53^ showed that the composition and variability of fish assemblages in upland streams in Kansas, USA, were strongly associated with distance from the nearest downstream reservoir.

Some studies have demonstrated that invasive species can induce shifts in species assemblages^54^ and promote homogenization of regional biotas^55–58^. Impoundments can facilitate invasion by non-native fishes^59^. However, Franssen and Tobler^25^ found that the shift in fish assemblage structure above a reservoir was influenced by changes in relative abundances of native species rather than invasion by non-native fishes. In the upper Yangtze River, shifts in assemblage structures resulted primarily from changes in native fish species. In this system, non-native fishes are much more prevalent within lacustrine habitats within the TGR^37,39^ than channel reaches upstream from the reservoir. *Coreius guichenoti* and *Pelteobagrus vachelli* were the dominant native species before impoundment. After impoundment, *Coreius guichenoti* declined, especially in the Mudong reach located near the margin of the reservoir where habitat shifted toward lentic conditions. This was consistent with the prediction by Park *et al*.^60^ that *Coreius guichenoti* would be at high risk of extinction after TGR filling.

Our results indicated that the functional structure of fish assemblages in the upper Yangtze River was altered after impoundment. In particular, we found that relative abundances of lotic-adapted species and periodic strategists declined in the Mudong reach after the filling of TGR. As environment in this reach shifted from lotic to lentic conditions and the abundance of the fluvial specialists declined, the abundance of lentic-adapted fishes increased greatly. In addition to transitioning to more lentic conditions, the Mudong reach now experiences fluctuations in water level, including daily changes, in response to dam operations for generating hydropower. Periodic strategists declined in the upper Yangtze River after impoundment, while opportunistic strategists became dominant. This finding is consistent with findings from research on regulated rivers in North America^61,62^.

Delariva *et al*.^63^ concluded that trophic interactions have a strong influence on the structure and dynamics of fish assemblages of subtropical rivers. Wang *et al*.^64^ found that altered hydrological regimes after TGD completion resulted in an immediate impact on the trophic structure of fish assemblages within the TGR. The density and biomass of benthic macroinvertebrates declined as water depth increased within the transitional zone of the TGR after impoundment^65^. Reductions in lotic-adapted aquatic macroinvertebrates that are important food resources for invertivorous fishes probably contributed to lower abundance of this trophic guild in the Mudong reach after impoundment. Several studies have reported rapid increases in the abundance of piscivorous fishes in newly formed reservoirs^66–69^, and this was attributed to a rapid increase in abundance of small prey fishes^62^. In the present study, the post-impoundment increase in the relative abundance of piscivores, particularly cyprinids in the subfamily Culterinae, was accompanied by increases in abundance of small opportunistic species that are prey for these fishes.

Even though the Hejiang and Yibin reaches are located well upstream from the TGR and retain lotic conditions, their fish assemblages nonetheless shifted following impoundment. In terms of functional groups, this shift was influenced by reductions in the relative abundance of fishes that were lotic-adapted, invertivorous, and/or periodic strategists. This shift in functional composition is the same as the pattern observed in Mudong where there was a transition from lotic to relatively lentic conditions. In all three reaches, there was a marked decline in abundance of a dominant invertivore, *Coreius guichenoti*. Studies from other regions have reported the influence of fish movement on shifts in local assemblage structure following damming. Schlosser^70^ found that dispersal significantly influenced dynamics of fish assemblages in temperate streams dammed by beavers (*Castor canadensis*). Antonio *et al*.^71^ captured and tagged migratory fishes below a major dam on the Upper Paraná River, Brazil, and translocated them into the reservoir. They discovered that the fish migrated to lotic habitats both downstream and upstream of the reservoir, presumably in avoidance of lentic conditions within the reservoir. In the Yangtze system, the abundance of several lotic-adapted species (e.g., *Rhinogobio cylindricus*) increased greatly within reaches located upstream after the filling of the TGR. This pattern could have resulted from migration from the reservoir in search of suitable lotic habitat. Piscivorous species that increased in abundance within these upstream reaches may have migrated there to exploit these small fishes. Overall, lotic-adapted species declined in the Hejiang and Mudong reaches after the filling of the TGR. In contrast to the pattern observed in the Hejiang and Mudong reaches, relative abundance of fishes categorized as members of the lotic guild increased in the Yinbin reach. This trend in the latter reach was largely influenced by species such as *Xenophysogobio boulengeri* and *Jinshaia sinensis*. Local populations of these native fishes might have experienced a release from competition when the formerly dominant and larger invertivore, *Coreius guichenoti*, declined in abundance following reservoir filling.

In summary, the impoundment formed by the TGD significantly affected fish assemblages in upstream reaches of the Yangtze River, as evidenced by the timing of regime shifts revealed by our analysis. Further support for this conclusion is the shift in functional structure of the assemblages. Changes in fish assemblage structure also could have been influenced by climate change or overfishing. However, at present, we have no data to relate observed shifts in fish assemblages to these drivers. We suggest that monitoring fish stocks in the upper Yangtze River should be continued in order to produce datasets capable of revealing ecological responses to ongoing environmental changes in the river and its watersheds. Given the large number of existing dams and plans for new hydropower dams in China and other regions supporting high fish diversity^7^, effective conservation requires improved understanding of the factors driving fish population and assemblage dynamics.

## Methods

### Ethics statement

All methods used in this study were conducted in accordance with the Laboratory Animal Management Principles of China. All experimental protocols in this study were approved by the Ethics Committee for Animal Experiments of the Institute of Hydrobiology, Chinese Academy of Sciences.

### Study area and fish surveys

The Yangtze River is more than 6,380 km long, which ranks it among the longest rivers in the word. The Yangtze originates from snow-capped Geladandong Mountain in the Tanggula Range, southwestern Qinhai Province, China, flows eastward, and discharges into the East China Sea. We surveyed fishes in the Yibin, Hejiang, and Mudong reaches. The Yibin site is located 299 river km (rkm) upstream from the inundated area created by a water level of 175 m ASL in the TGR, Hejiang is 97 rkm upstream from the inundated area, and the Mudong site is located 85 rkm downstream from the inundated area (Fig. 1). Based on flow characteristics^72^, these survey sites represent riverine (Yibin and Hijiang) and transitional (Mudong) habitats in the main channel of the Yangtze River.

We sampled fishes in the three reaches during two seasons (May- June, September- October) each year from 1997 to 2015. However, sampling was not conducted in 2003 and 2004 at Hejiang and Yibin or in 2003–2005 at Mudong because of a governmental travel ban that was imposed to combat the outbreak of Severe Acute Respiratory Syndromes (SARS). Each survey was conducted for 15–20 days within each season of each year, with 20–30 km surveyed within each reach.

#### Surveys aided by local fishers

Each survey was done using at least two local fishing boats at each location. Because fishermen had their own methods of fishing based on their experience, multiple fishing methods were employed during each survey^73,74^. Fishermen preferred to capture fish in the area where they could obtain more fish. When their catch rate declines, they moved to a different area. Therefore, we assumed that their effort is distributed according to the availability of fish within various habitats at each location. Because effort varied among time intervals, we analyzed species relative abundance (species proportions) in this study (see Data analysis).

Mid-channel habitats: To catch fish in the mid-channel, fishermen used three types of fishing gear: drifting gill nets (net height ranged from 1–2.3 m; length ranged from 50–80 m; combinations of mesh sizes: 1–14 cm), multi-cod-end seines (150 m long × 1.5 m high; mesh sizes = 1, 1.5, or 2 cm; cod ends = 500–800), and trawl nets (net opening = 4.5 m × 1.8 m; net depth = 8 m; mesh size = 1 or 2 cm). Drifting gill nets effectively captured fish from positions near the bottom and low in the water column, and seines and trawl nets were effective in catching fish from middle-upper levels of the water column. Each of these fishing gears was deployed approximately every two hours during a 12-h period during each day of the survey period. The total fishing effort per survey period/site was from 29 to 157 boat-days in Mudong reach, from 18 to 158 boat-days in Hejiang reach, and from 13 to 165 boat-days in Yibin reach. Greater effort was required to capture fishes from mid-channel habitats when discharge, depth and flow velocity were greater.

Near-shore habitats: During each survey at each site, four types of fishing gear were used in near-shore areas: stationary gill nets (35 m long × 5 m high, with mesh sizes of 1, 2, 3, 4, 5, 6, 10, 11 cm), hoop nets (mesh sizes of 0.5, 1, 1.5 cm), and trotlines (200–1,900 hooks per line) baited with earth worms, meal worms (the larval of *Tenebrio molitor*), or an artificial bait. Gill nets and trotlines effectively captured fish from all layers of the water column, and hoop nets captured fish near the bottom. These fishing gears were set at 0600 h and retrieved them at 0600 h the following day. During each survey period, sampling effort in near-shore habitats varied according to river flow conditions, and was from 1 to 39 boat-days in Mudong reach, from 3 to 106 boat-days in Hejiang reach, from 14 to 105 boat-days in Yibin reach. When discharge was higher, more effort was required to achieve comprehensive samples of fishes present in near-shore habitats. To capture rare species that may have avoided gear types used by local fishers, we employed four additional fishing methods: boat electrofishing, lift net, cast net, and trap net during each survey period at each sampling site. We did not obtain samples from each of these gear types during each day of each survey.

Fish specimens were counted and identified to species based on identification guidance described by Ding^75^. The data were converted into species proportional abundance (i.e., relative abundance) by taking the number of specimens of a given species and dividing it by the total number of specimens captured during that survey period for that site.

**Figure 5:**
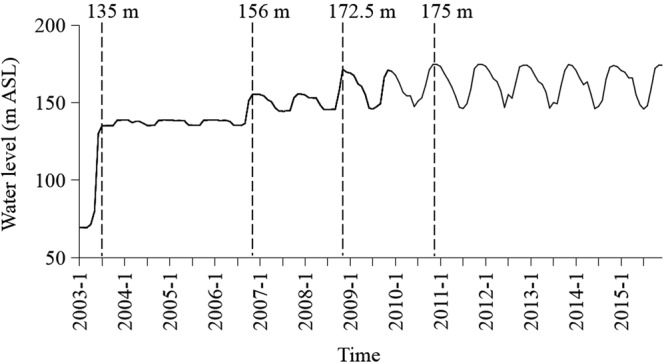
Water level of the Three Gorges Reservoir from 2003–2015. Dotted lines separate reservoir filling stages.

Water level data for the TGR were obtained from China Three Gorges Project Corporation (Fig. 5).

### Data analysis

The relative abundance data were compiled to create a time series for each species at each location. Species with relative abundance greater than 10% were defined as dominant species. Using the relative abundance data for all species, we performed principal components analysis (PCA) to ordinate fish assemblage of each study reach during different periods from 1997 to 2015. We estimated the time of regime shift in the fish assemblage in the three reach by using STARS (Sequential t-test Analysis for Regime Shift detection)^76,77^. The first and second scores (PC1 and PC2) in the three reaches were analyzed with STARS, which estimates breakpoints that mark the first year of each shift in assemblage structure using the Student t-test. The values of breakpoints are significantly different to the mean of the previous regime. The following parameters were set before running the STARS model. The significance level was set at 0.05 for all statistical tests. A cut-off interval of 10 years was set as the minimum length of a regime. The Huber weight, which was set at 1, improved the analysis by putting less weight on outliers.

Non-metric multidimensional scaling (nMDS) was used to estimate the distributions of similarities among fish assemblages of the three reaches for each year from 1997 to 2015 based on Bray-Curtis distance. Stress values < 0.20 were considered to reflect acceptable goodness-of-fit^78^. PERMANOVA with Bray-Curtis distance (9999 permutation) was used to test for differences in the fish assemblages in each reach before and after a regime shift^79^. Using the method of Tilman^80^, fish abundance data were used to calculate the temporal stability index of each local assemblage before and after a regime shift. This analysis produces an index of stability, with higher values indicating greater stability.

Three diversity indices (Simpson, Shannon-Wiener, and Buzas & Gibson’s evenness index) were calculated and compared between pre and post-shift using the Student t-test. We also analyzed ecological attributes (habitat, trophic, life history) to assess changes in assemblage functional structure in relation to the impoundment^81^. Fish species were classified into functional groups based on information reported in previous studies. Based on information in Gao *et al*.^37^, species were assigned to one of three habitat categories: lotic, lentic, and generalist. Trophic categories were herbivore, invertivore, omnivore and piscivore, with species assignments mainly based on adult stages and information reported by Ding and Liu^82^. Life history categories included periodic, equilibrium, and opportunistic strategists based on the theory of life-history strategies proposed by Winemiller^83^ and Winemiller and Rose^84^. Species assignment to life history categories was based on information reported by Li^85^. The matrix of fish species and relative abundance in the three reaches was compared using SIMPER (Similarity percentages), a dissimilarity test that determines the degree that species and functional groups contribute to the differences in assemblage structure. Here we compared differences within each reach before and after completion of the TGD. SIMPER analyzes species that contribute to Bray-Curtis dissimilarities or Euclidean distances between groups of samples^78^.

Computation of PCA, nMDS, PERMANOVA, SIMPER, and diversity indices was performed with PAST 3.15 software^86^. STARS was performed using the Sequential Regime Shift Detection program^87^, which was available as a Word Processing System (WPS) Table add-in (Kingsoft Corporation Limited)^88^. Community stability indices were computed with R 3.3.3^89^ using the package ‘codyn’.

## Supplementary information

Supplementary Information

## Data Availability

The datasets generated during and/or analyzed during the current study are available from the corresponding author upon reasonable request.

## References

[1] Rosenberg DM (1997). Large-scale impacts of hydroelectric development. Environ. Res..

[2] Poff NL (1997). The natural flow regime. BioScience.

[3] Poff NL, Olden JD, Merritt DM, Pepin DM (2007). Homogenization of regional river dynamics by dams and global biodiversity implications. Proc. Natl. Acad. Sci. USA.

[4] Poff NL, Hart DD (2002). How dams vary and why it matters for the emerging science of dam removal. BioScience.

[5] Fearnside PM (2006). Dams in the Amazon: Belo Monte and Brazil’s hydroelectric development of the Xingu River Basin. Environ. Manage..

[6] Ziv G, Baran E, Nam S, Rodríguez-Iturbe I, Levin SA (2012). Trading-off fish biodiversity, food security, and hydropower in the Mekong River Basin. Proc. Natl. Acad. Sci. USA.

[7] Winemiller KO (2016). Balancing hydropower and biodiversity in the Amazon, Congo, and Mekong. Science.

[8] Williams, G. P., & Wolman, M. G. *Downstream effects of dams on alluvial rivers*. (United States Government Printing Office, 1984).

[9] Kondolf GM (1997). PROFILE: hungry water: effects of dams and gravel mining on river channels. Environ. Manage..

[10] Freitas, C. E. C. *et al*. The potential impacts of global climatic changes and dams on Amazonian fish and their fisheries *in New Advances and Contributions to Fish Biology* (ed. Turker H.) 176–195 (InTech, Rijeka, Croatia, 2012).

[11] Pelicice FM, Pompeu PS, Agostinho AA (2015). Large reservoirs as ecological barriers to downstream movements of Neotropical migratory fish. Fish Fish..

[12] Baxter RM (1977). Environmental effects of dams and impoundments. Annu. Rev. Ecol. Syst..

[13] Dynesius, M. & Nilsson, C. Fragmentation and flow regulation of river systems in the northern third of the world. *Science* 753–753 (1994). 10.1126/science.266.5186.753 17730396

[14] Downing JA (2006). The global abundance and size distribution of lakes, ponds, and impoundments. Limnol. Oceanogr..

[15] Liermann CR, Nilsson C, Robertson J, Ng RY (2012). Implications of dam obstruction for global freshwater fish diversity. BioScience.

[16] Poff NL, Zimmerman JK (2010). Ecological responses to altered flow regimes: a literature review to inform the science and management of environmental flows. Freshwater Biol..

[17] Greathouse EA, Pringle CM, McDowell WH, Holmquist JG (2006). Indirect upstream effects of dams: consequences of migratory consumer extirpation in Puerto Rico. Ecol. Appl..

[18] Bishop KA, Bell JD (1978). Observations on the fish fauna below Tallowa Dam (Shoalhaven River, New South Wales) during river flow stoppages. Mar. Freshw. Res..

[19] Kanehl PD, Lyons J, Nelson JE (1997). Changes in the habitat and fish community of the Milwaukee River, Wisconsin, following removal of the Woolen Mills Dam. North Am. J. Fish. Manage..

[20] Cumming GS (2004). The impact of low-head dams on fish species richness in Wisconsin, USA. Ecol. Appl..

[21] de Mérona B, Dos Santos GM, De Almeida RG (2001). Short term effects of Tucuruí Dam (Amazonia, Brazil) on the trophic organization of fish communities. Environ. Biol. Fishes.

[22] de Mérona B, Vigouroux R, Tejerina-Garro FL (2005). Alteration of fish diversity downstream from Petit-Saut Dam in French Guiana. Implication of ecological strategies of fish species. Hydrobiologia.

[23] Mims MC, Olden JD (2013). Fish assemblages respond to altered flow regimes via ecological filtering of life history strategies. Freshw. Biol..

[24] Taylor JM, Seilheimer TS, Fisher WL (2014). Downstream fish assemblage response to river impoundment varies with degree of hydrologic alteration. Hydrobiologia.

[25] Franssen NR, Tobler M (2013). Upstream effects of a reservoir on fish assemblages 45 years following impoundment. J. Fish Biol..

[26] Olden, J. D. Challenges and opportunities for fish conservation in dam-impacted waters *in Conservation of Freshwater Fishes* (eds Closs, G. P., Krkosek, M. & Olden, J. D.) 107–148 (Cambridge University Press, 2015).

[27] Lima AC, Sayanda D, Soares AM, Wrona FJ, Monaghan KA (2016). Integrating taxonomic and trait analyses to assess the impact of damming on fish communities in a northern cold region river. Can. J. Fish. Aquat. Sci..

[28] Lopez-Pujol J, Ren MX (2009). Biodiversity and the Three Gorges Reservoir: a troubled marriage. J. Nat. Hist..

[29] Fu BJ (2010). Three Gorges Project: efforts and challenges for the environment. Prog. Phys. Geogr..

[30] Zhang M, Shao M, Xu Y, Cai Q (2010). Effect of hydrological regime on the macroinvertebrate community in Three-Gorges Reservoir, China. Quat. Int..

[31] Zheng T, Mao J, Dai H, Liu D (2011). Impacts of water release operations on algal blooms in a tributary bay of Three Gorges Reservoir. Sci. China Technol. Sc..

[32] Gao X, Li MZ, Lin PC, Duan ZH, Liu HZ (2013). Environmental cues for natural reproduction of the Chinese sturgeon, *Acipenser sinensis* Gray, 1835, in the Yangtze River, China. J. Appl. Ichthyol..

[33] Gao X, Lin P, Li M, Duan Z, Liu H (2014). Effects of water temperature and discharge on natural reproduction time of the Chinese Sturgeon, *Acipenser sinensis*, in the Yangtze River, China and impacts of the impoundment of the Three Gorges Reservoir. Zool Sci..

[34] Gao X, Lin P, Li M, Duan Z, Liu H (2016). Impact of the Three Gorges Dam on the spawning stock and natural reproduction of Chinese sturgeon in Changjiang River, China. Chin. J. Oceanol. Limnol..

[35] Li M, Duan Z, Gao X, Cao W, Liu H (2016). Impact of the Three Gorges Dam on reproduction of four major Chinese carps species in the middle reaches of the Changjiang River. Chin. J. Oceanol. Limnol..

[36] Wu J (2004). The three gorges dam: an ecological perspective. Front. Ecol. Environ..

[37] Gao X, Zeng Y, Wang J, Liu H (2010). Immediate impacts of the second impoundment on fish communities in the Three Gorges Reservoir. Environ. Biol. Fishes.

[38] Yang S, Gao X, Li M, Ma B, Liu H (2012). Interannual variations of the fish assemblage in the transitional zone of the Three Gorges Reservoir: persistence and stability. Environ. Biol. Fishes.

[39] Gao X, Liu H (2016). Current fish resources in the upper reach of the Yangtze River and conservation strategies. Am. Fish. Soc. Symp..

[40] Kirkman SP (2015). Regime shifts in demersal assemblages of the Benguela Current Large Marine Ecosystem: a comparative assessment. Fish. Oceanogr..

[41] Scheffer M, Carpenter S, Foley JA, Folke C, Walker B (2001). Catastrophic shifts in ecosystems. Nature.

[42] Scheffer M, Carpenter SR (2003). Catastrophic regime shifts in ecosystems: linking theory to observation. Trends Ecol. Evol..

[43] Biggs R, Carpenter SR, Brock WA (2009). Turning back from the brink: detecting an impending regime shift in time to avert it. Proc. Natl. Acad. Sci. USA.

[44] Reid PC, de Fatima Borges M, Svendsen E (2001). A regime shift in the North Sea circa 1988 linked to changes in the North Sea horse mackerel fishery. Fish. Res..

[45] Jiao Y (2009). Regime shift in marine ecosystems and implications for fisheries management, a review. Rev. Fish Biol. Fish..

[46] de Tezanos Pinto P, O’Farrell I (2014). Regime shifts between free-floating plants and phytoplankton: a review. Hydrobiologia.

[47] Morse RE, Friedland KD, Tommasi D, Stock C, Nye J (2017). Distinct zooplankton regime shift patterns across ecoregions of the US Northeast continental shelf Large Marine Ecosystem. J. Mar. Syst..

[48] Yletyinen J (2016). Regime shifts in marine communities: a complex systems perspective on food web dynamics. Proc. R. Soc,. B.

[49] Beaugrand G (2004). The North Sea regime shift: evidence, causes, mechanisms and consequences. Prog. Oceanogr..

[50] Möllmann C, Müller-Karulis B, Kornilovs G, St John MA (2008). Effects of climate and overfishing on zooplankton dynamics and ecosystem structure: regime shifts, trophic cascade, and feedback loops in a simple ecosystem. ICES J. Mar. Sci..

[51] Auber A, Travers-Trolet M, Villanueva MC, Ernande B (2015). Regime shift in an exploited fish community related to natural climate oscillations. PloS one.

[52] Röpke CP (2017). Simultaneous abrupt shifts in hydrology and fish assemblage structure in a floodplain lake in the central Amazon. Sci. Rep..

[53] Falke JA, Gido KB (2006). Effects of reservoir connectivity on stream fish assemblages in the Great Plains. Can. J. Fish. Aquat. Sci..

[54] Andersen T, Carstensen J, Hernandez-Garcia E, Duarte CM (2009). Ecological thresholds and regime shifts: approaches to identification. Trends Ecol. Evol..

[55] McKinney ML, Lockwood JL (1999). Biotic homogenization: a few winners replacing many losers in the next mass extinction. Trends Ecol. Evol..

[56] Rahel FJ (2002). Homogenization of freshwater faunas. Annu. Rev. Ecol. Syst..

[57] Olden JD, Poff NL, Douglas MR, Douglas ME, Fausch KD (2004). Ecological and evolutionary consequences of biotic homogenization. Trends Ecol. Evol..

[58] Olden JD (2006). Biotic homogenization: a new research agenda for conservation biogeography. J. Biogeogr..

[59] Johnson PT, Olden JD, Vander Zanden MJ (2008). Dam invaders: impoundments facilitate biological invasions into freshwaters. Front. Ecol. Environ..

[60] Park YS, Chang J, Lek S, Cao W, Brosse S (2003). Conservation strategies for endemic fish species threatened by the Three Gorges Dam. Conserv. Biol..

[61] Olden JD, Poff NL, Bestgen KR (2006). Life-history strategies predict fish invasions and extirpations in the Colorado River Basin. Ecol. Monogr..

[62] Mims MC, Olden JD (2012). Life history theory predicts fish assemblage response to hydrologic regimes. Ecology.

[63] Delariva RL, Hahn NS, Kashiwaqui EAL (2013). Diet and trophic structure of the fish fauna in a subtropical ecosystem: impoundment effects. Neotrop. Ichthyol..

[64] Wang J, Li L, Xu J, Gu B (2016). Initial response of fish trophic niche to hydrological alteration in the upstream of three gorges dam. Ecol. Proc..

[65] Wang B, Liu X, Peng Z, Yang Z (2015). The community structure of zoobenthos in the Three Gorges Reservoir: a comparison before and after the impoundment. Acta Hydrobiologica Sinica.

[66] Petrere M (1996). Fisheries in large tropical reservoirs in South America. Lakes Reserv. Res. Manage..

[67] Gomes LC, Miranda LE (2001). Riverine characteristics dictate composition of fish assemblages and limit fisheries in reservoirs of the Upper Paraná River Basin. River Res. Appl..

[68] Luz-Agostinho KD (2006). Food spectrum and trophic structure of the ichthyofauna of Corumbá reservoir, Paraná river Basin, Brazil. Neotrop. Ichthyol..

[69] Mol JH, Mérona BD, Ouboter PE, Sahdew S (2007). The fish fauna of Brokopondo Reservoir, Suriname, during 40 years of impoundment. Neotrop. Ichthyol..

[70] Schlosser IJ (1995). Dispersal, boundary processes, and trophic-level interactions in streams adjacent to beaver ponds. Ecology.

[71] Antonio RR (2007). Blockage of migration routes by dam construction: can migratory fish find alternative routes?. Neotrop. Ichthyol..

[72] Thornton, K. W., Kimmel, B. L., & Payne, F. E. *Reservoir limnology: ecological perspectives*. (John Wiley & Sons, 1990).

[73] Hubert, W. A., Pope, K. L. & Dettmers, J. M. Passive capture techniques *in Fisheries techniques, 3rd edition*. (eds Zale, A. V., Parrish, D. L., & Sutton, T. M.) 223–265 (American Fisheries Society, 2012).

[74] de Paiva Affonso I, Gomes LC, Agostinho AA, Latini JD, García-Berthou E (2016). Interacting effects of spatial gradients and fishing gears on characterization of fish assemblages in large reservoirs. Rev. Fish Biol. Fish..

[75] Ding, R. H. *The fishes of Sichua*n. (Sichuan Publishing House of Science and Technology, 1994).

[76] Rodionov SN (2004). A sequential algorithm for testing climate regime shifts. Geophys. Res. Lett..

[77] Rodionov SN (2006). Use of prewhitening in climate regime shift detection. Geophys. Res. Lett..

[78] Clarke, K. R. & Warwick, R. M. *Change in marine communities: An approach to statistical analysis and interpretation, 2*^*nd*^*edition*. (PRIMER-E Ltd, 2001).

[79] Anderson MJ (2001). A new method for non-parametric multivariate analysis of variance. Austral ecol..

[80] Tilman D (1999). The ecological consequences of changes in biodiversity: a search for general principles. Ecology.

[81] Hoeinghaus DJ, Winemiller KO, Birnbaum JS (2007). Local and regional determinants of stream fish assemblage structure: inferences based on taxonomic vs. functional groups. J. Biogeogr..

[82] Ding B, Liu H (2011). Analysis of the fish feeding guild composition in the Yangtze River. Sichuan Journal of Zoology.

[83] Winemiller, K. O. Life-history strategies and the effectiveness of sexual selection. *Oikos* 318–327 (1992).

[84] Winemiller KO, Rose KA (1992). Patterns of life-history diversification in North American fishes: implications for population regulation. Can. J. Fish. Aquat. Sci..

[85] Li, M. *Study on the life history strategies of fishes in the Yangtze River and its adaption to environment during early life history stage*. (University of Chinese Academy of Sciences, 2012).

[86] Hammer, Ř., Harper, D. A. T. & Ryan, P. D. PAST: Paleontological Statistics Software Package for Education and Data Analysis-Palaeontol. http://palaeo-electronica.org/2001_1/past/issue1_01.htm (2001).

[87] Rodionov, S. N. Sequential regime shift detectionm software, version 3.2. https://www.beringclimate.noaa.gov/regimes/ (2006).

[88] Kingsoft Corporation Limited. User manual of spreadsheet 2016. www.wps.com (2016).

[89] R Development Core Team. R: A language and environment for statistical computing. R Foundation for Statistical Computing, Vienna, Austria. ISBN 3-900051-07-0 (2014).

