# Supplementary material for: Regime shift in fish assemblage structure in the Yangtze River following construction of the Three Gorges Dam
Source: Sci Rep. 2019 Mar 12;9:4212. doi: 10.1038/s41598-019-38993-x (PMC6414653; doi:10.1038/s41598-019-38993-x)
Supplement: Supplementary file 1 — Supplementary Information [file 41598_2019_38993_MOESM1_ESM.pdf]

Supplementary Information

Regime shift in fish assemblage structure in the Yangtze River following construction of the Three Gorges Dam

Xin Gao[1], Masami Fujiwara[2], Kirk O. Winemiller[2], Pengcheng Lin[1], Mingzheng Li[1], Huanzhang Liu[1,*]

[1] Key Laboratory of Aquatic Biodiversity and Conservation of Chinese Academy of Sciences, Institute of Hydrobiology, Chinese Academy of Sciences, Wuhan, Hubei, China

[2] Department of Wildlife and Fisheries Sciences, Texas A&M University, College Station, TX, 77843-2258, USA.

[*]Corresponding. hzliu@ihb.ac.cn

Table S1 Fish species list in Mudong, Hejiang, and Yibin reaches from 1997 to 2015

No.	Species	Mudong		Hejiang		Yibin		Nonative species	Total abundance (individual)
		Pre-shift	Post-shift	Pre-shift	Post-shift	Pre-shift	Post-shift		
1	*Acipenser dabryanus*	+			+		+		10
2	*Acipenser schrenckii*		+					+	2
3	hybrid sturgeon		+		+			+	3
4	*Anguilla japonica*				+	+			2
5	*Myxocyprinus asiaticus*		+	+	+	+	+		87
6	*Zacco platypus*	+	+		+	+	+		100
7	*Opsariichthys bidens*		+		+	+			40
8	*Aphyocypris chinensis*			+					2
9	*Mylopharyngodon piceus*		+		+				14
10	*Ctenopharyngodon idellus*	+	+	+	+	+	+		460
11	*Tinca tinca*				+	+		+	7
12	*Squaliobarbus curriculus*	+	+	+	+				61
13	*Elopichthys bambusa*	+	+						9
14	*Sinibrama macrops*			+		+	+		10
15	*Sinibrama taeniatus*			+	+				8
16	*Ancherythroculter wangi*	+	+	+	+				11
17	*Ancherythroculter kurematsui*	+	+	+	+	+			264
18	*Ancherythroculter nigrocauda*	+	+	+	+				6
19	*Pseudolaubuca sinensis*	+	+	+	+	+	+		1401
20	*Pseudolaubuca engraulis*	+	+	+	+	+	+		2696
21	*Hemiculter leucisculus*	+	+	+	+	+	+		3386

22	*Hemiculter bleekeri*	+	+	+	+	+	+		7548
23	*Hemiculter tchangi*	+	+	+	+	+			1294
24	*Hemiculterella sauvagei*		+						1
25	*Tor* (*Folifer*) *brevifilis brevifilis*	+		+					9
26	*Cultrichthys erythropterus*		+	+	+	+	+		150
27	*Culter alburnus*	+	+	+	+	+	+		1063
28	*Culter mongolicus mongolicus*	+	+	+	+		+		806
29	*Culter dabryi*	+	+						410
30	*Culter oxycephalus*	+	+	+					4
31	*Culter oxycephaloides*	+	+	+					10
32	*Parabramis pekinensis*	+	+	+	+				16
33	*Megalobrama pellegrini*	+	+				+	+	27
34	*Megalobrama pellegrini*	+	+	+	+	+	+		276
35	*Megalobrama amblycephala*		+	+	+	+	+	+	22
36	*Xenocypris argentea*	+	+		+				16
37	*Xenocyprisdavidi*	+	+	+	+				273
38	*Xenocypris yunnanensis*			+		+			7
39	*Xenocypris fangi*				+				3
40	*Xenocypris microlepis*			+	+				11
41	*Pseudobrama simoni*	+	+	+	+	+	+		3258
42	*Hypophthalmichthys molitrix*	+	+	+	+	+	+		378
43	*Hypophthalmichthys nobilis*		+	+	+		+		182
44	*Hemibarbus labeo*	+	+	+	+	+	+		391
45	*Hemibarbus maculatus*	+	+	+	+	+	+		1817

46	*Pseudorasbora parva*	+	+	+	+	+	+	822
47	*Sarcocheilichthys sinensis*	+	+	+	+			62
48	*Sarcocheilichthys nigripinnis*		+	+	+			25
49	*Gnathopogon herzensteini*				+			77
50	*Gnathopogon imberbis*	+			+			41
51	*Squalidus argentatus*	+	+	+	+	+	+	23087
52	*Squalidus wolterstorffi*	+	+					22
53	*Coreius heterodon*	+	+	+	+	+	+	27992
54	*Coreius guichenoti*	+	+	+	+	+	+	137471
55	*Rhinogobio typus*	+	+	+	+	+	+	5201
56	*Rhinogobio cylindricus*	+	+	+	+	+	+	19920
57	*Rhinogobio ventralis*	+	+	+	+	+	+	20219
58	*Platysmacheilus exiguus*		+					1
59	*Platysmacheilus nudiventris*		+	+	+	+	+	877
60	*Abbottina rivularis*	+	+	+	+	+	+	576
61	*Abbottina obtusirostris*			+				75
62	*Microphysogobio kiatingensis*	+	+		+			582
63	*Pseudogobio vaillanti*	+	+		+			38
64	*Saurogobio dumerili*			+				21
65	*Saurogobio dabryi*	+	+	+	+	+	+	33653
66	*Saurogobio gymnocheilus*	+	+	+	+	+		2205
67	*Gobiobotia* (*Gobiobotia*) *filifer*	+	+	+	+	+	+	14117
68	*Xenophysogobio boulengeri*	+	+	+	+	+	+	9675
69	*Xenophysogobio nudicorpa*		+		+	+	+	437

70	*Rhodeus ocellatus*	+	+		+	+	+	1070
71	*Rhodeus fangi*		+					84
72	*Rhodeus sinensis*	+	+	+	+	+		1053
73	*Acheilognathus macropterus*	+	+	+	+			514
74	*Acheilognathus omeiensis*	+			+			4
75	*Acheilognathus chankaensis*		+					12
76	*Spinibarbus sinensis*	+	+	+	+		+	563
77	*Acrossocheilus monticolus*		+		+			4
78	*Acrossocheilus yunnanensis*				+			1
79	*Onychostoma sima*	+	+	+	+	+	+	131
80	*Bangana rendahli*	+	+	+				10
81	*Pseudogyrinocheilus procheilus*	+	+		+	+	+	21
82	*Garra pingi pingi*	+		+	+	+	+	103
83	*Schizothorax* (*Schizothorax*) *chongi*			+	+	+		6
84	*Schizothorax* (*Schizothorax*) *wangchiachii*				+		+	5
85	*Schizothorax* (*Schizothorax*) *prenanti*					+		7
86	*Procypris rabaudi*	+	+	+	+	+	+	418
87	*Cyprinus* (*Cyprinus*) *carpio*	+	+	+	+	+	+	2386
88	*Carassius auratus*	+	+	+	+	+	+	5668
89	*Lepturichthys fimbriata*	+	+	+	+	+	+	4093
90	*Jinshaia abbreviata*	+		+	+			97
91	*Jinshaia sinensis*	+	+	+	+	+	+	4567
92	*Sinogastromyzon sichangensis*			+	+			42

93	*Sinogastromyzon szechuanensis szechuanensis*		+	+	+	+		392
94	*Paracobitis variegatus*		+	+	+	+	+	815
95	*Paracobitis potanini*			+	+	+	+	552
96	*Paracobitis wujiangensis*		+		+			241
97	*Yunnanilus sichuanensis*				+			140
98	*Botia reevesae*	+	+	+	+	+		119
99	*Botia superciliaris*	+	+	+	+	+	+	17685
100	*Parabotia fasciata*	+	+		+	+	+	79
101	*Parabotia bimaculata*		+		+			41
102	*Leptobotia elongata*	+	+	+	+	+	+	6162
103	*Leptobotia taeniops*	+	+	+	+	+	+	692
104	*Leptobotia pellegrini*	+	+	+	+	+	+	412
105	*Leptobotia microphthalma*		+		+			154
106	*Leptobotia rubrilabris*	+	+	+	+	+	+	1512
107	*Cobitis sinensis*	+	+		+			352
108	*Misgurnus anguillicaudatus*	+	+	+	+	+	+	240
109	*Paramisgurnus dabryanus*		+	+	+			19
110	*Pelteobagrus fulvidraco*	+	+	+	+	+	+	1059
111	*Pelteobagrus eupogon*	+	+		+			22
112	*Pelteobagrus vachelli*	+	+	+	+	+	+	77917
113	*Pelteobagrus nitidus*	+	+	+	+	+	+	11344
114	*Leiocassis longirostris*	+	+	+	+	+	+	4236
115	*Leiocassis crassilabris*	+	+	+	+	+	+	6741

116	*Pseudobagrus ussuriensis*			+		+			108
117	*Pseudobagrus pratti*	+		+	+	+	+		300
118	*Pseudobagrus truncatus*	+	+	+	+	+	+		1107
119	*Pseudobagrus emarginatus*	+	+	+	+	+	+		3097
120	*Pseudobagrus brevicaudatus*	+		+		+			13
121	*Mystus macropterus*	+	+	+	+	+	+		696
122	*Silurus asotus*	+	+	+	+	+	+		1121
123	*Silurus meridionalis*	+	+	+	+	+	+		2493
124	*Liobagrus marginatus*		+	+	+	+	+		304
125	*Liobagrus nigricauda*		+		+				533
126	*Liobagrus marginatoides*			+	+	+			206
127	*Glyptothorax fokiensis*	+	+	+	+	+	+		1505
128	*Glyptothorax sinensis*	+	+	+	+	+	+		2279
129	*Clarias fuscus*	+		+		+	+		80
130	*Clarias gariepinus*				+			+	1
131	*Ictalurus punctatus*			+	+		+	+	12
132	*Protosalanx chinensis*		+		+			+	78
133	*Salangichthys tangkahkeii*				+	+	+		62
134	*Hyporamphus intermedius*	+	+		+				8
135	*Oryzias latipes*				+	+			4
136	*Monopterus albus*					+			1
137	*Siniperca chuatsi*	+	+	+	+				340
138	*Siniperca scherzeri*	+	+	+	+				19
139	*Siniperca kneri*	+	+	+					47

140	*Channa argus*		+		+				4
141	*Micropercops swinhonis*		+	+	+	+			44
142	*Odontobutis obscurus*		+		+				8
143	*Mugilogobius myxodermus*				+				32
144	*Rhinogobius giurinus*	+	+	+	+	+	+		7893
145	*Rhinogobius cliffordpopei*				+				7
146	*Rhinogobius brunneus*		+		+				73
147	*Micropterus salmoides*		+					+	1
148	*Oreochromis sp.*		+		+			+	6
149	*Lucioperca lucioperca*				+		+	+	6
150	*Piaractus brachypomus*	+	+					+	3
